# Molecular diagnostic of cytomegalovirus, Epstein Barr virus and Herpes virus 6 infections among blood donors by multiplex real-time PCR in Ouagadougou, Burkina Faso

**DOI:** 10.11604/pamj.2016.24.298.6578

**Published:** 2016-08-03

**Authors:** Lassina Traore, Issoufou Tao, Cyrille Bisseye, Birama Diarra, Tegwindé Rebeca Compaore, Yacouba Nebie, Maleki Assih, Alice Ouedraogo, Theodora Zohoncon, Florencia Djigma, Djénéba Ouermi, Nicolas Barro, Mahamoudou Sanou, Rasmata Traore Ouedraogo, Jacques Simpore

**Affiliations:** 1 Biomolecular Research Center Pietro Annigoni Labiogene, UFR / SVT, University of Ouagadougou BP 364 Ouagadougou, Burkina Faso; 2 Laboratory of Molecular and Cellular Biology, University of Sciences and Techniques of Masuku, P 943 Franceville, Gabon; 3 Molecular Biology, Epidemiology and Monitoring of Communicable of Bacteria and Viruses Through Food, UFR / SVT, University of Ouagadougou, Burkina Faso

**Keywords:** EBV, CMV, HHV-6, Burkina Faso

## Abstract

**Introduction:**

In most developing countries, Cytomegalovirus (CMV), Epstein Barr virus (EBV) and Herpes virus 6 (HHV-6) are not diagnosed in blood donors. The aim of this study is to determine the prevalence of these viruses in blood donors from the city of Ouagadougou, Burkina Faso.

**Methods:**

The study included 198 blood donors of the Regional Blood Transfusion Centre of Ouagadougou. Multiplex real time PCR was used to diagnose the three viruses. Statistical analysis was performed with the software EpiInfo version 6 and SPSS version 17. P values ≤ 0.05 were considered significant.

**Results:**

Of 198 samples tested, 18 (9.1%) were positive to at least one of the three viruses. In fact, 10 (5.1%) were positive for EBV, 10 (5.1%) positive for CMV and 12 (6.1%) positive for HHV-6. Viral infections were higher in women than in men, EBV (8,6% versus 4.3%), CMV (8.6% versus 3.7%) and HHV-6 (11.4% versus 4.9%). EBV / CMV / HHV-6 co-infection was found in 3.5% (7/198) of blood donors.

**Conclusion:**

The prevalence recorded in this study is low compared to those found in previous studies from the sub-region among blood donors. The molecular diagnostic test used in our study could explain the differences with previous studies.

## Introduction

In Burkina Faso, as in most developing countries, Cytomegalovirus (CMV), Epstein Barr virus (EBV) and Herpes virus 6 (HHV-6) are not diagnosed in blood donors [1]. CMV, EBV and HHV-6 infections are transmitted through blood and remain a significant cause of mortality and morbidity, especially in immunocompromised persons [2, 3]. Gastrointestinal CMV infections are extremely common in immunosuppressed patients [4]. CMV infection has been considered an important resistance factor to therapy in patients with intestinal inflammation, taking antiviral therapy [5, 6]. CMV infection of the intestine is clinically significant but not always with the classic cytopathic viral changes [7]. Congenital CMV infection is cited as the most important cause of infectious diseases of the central nervous system and hearing loss in children. It also endangers human fetus development; this issue makes the development of new strategies to prevent the acquisition and transmission of CMV a priority [8, 9]. EBV is one of the most common human viruses and is found throughout the world. It has been reported that 90% of adults are asymptomatic carriers. In immunocompromised individuals, the virus is activated and becomes an oncogene. EBV early infection has been described in children in Africa but also in developed countries where the primary infection is delayed until adulthood [10–12]. HHV6 is a virus similar to CMV; its discovery dates back to1986. It spread on the pandemic mode as CMV and EBV. An estimated of 80 to 90% is the number of people carrying the HHV-6 intermittently in their saliva [13, 14]. These three viruses are responsible for asymptomatic infections in immunocompetent individuals. In fact they cause disease especially in patients whose immune systems have been weakened, such as those treated with immunosuppressors, those affected by the human immunodeficiency virus (HIV) [15] and pregnant women. These viruses are responsible for many cancers, including Burkit's lymphoma (BL) observed endemic among African children [16], the nasopharynx lymphoma [17], B and T cells lymphomas [11, 18]. These types of cancers called non-Hodgkin represent the third type of cancer in sub-Saharan Africa, after breast and cervical cancers [19]. The objective of this study was to determine the prevalence of these three viruses in blood donors of the Regional Blood Transfusion Centre of Ouagadougou (CRTSO).

## Methods

**Sampling:** This cross-sectional study was conducted in february 2012 at the National Blood Transfusion Centre of Ouagadougou in Burkina Faso. Two hundred (200) apparently healthy individuals aged ≤17 years with a weight >50 kg were recruited for blood donation. All donors answered questions intending to exclude individuals having signs of hepatitis, pregnant women and people having experienced high-risk sexual behaviour within 2 weeks preceding the intended donation.

**DNA extraction and amplification of CMV, EBV and HHV-6:** The genomic DNA of the viruses was extracted by using the extraction commercial kit DNA-Sorb-B (Sacace Biotechnologies, Como, Italy) following the manufacturer´s instructions. The amplification of CMV, EBV and HHV-6 was made by multiplex real time PCR using the amplification kit CMV / EBV / HHV-6 as Real-TM (Sacace Biotechnology, Italy) with the SaCycler-96 Real-Time PCR V.7.3 (Sacace Biotechnology, Como, Italy).

**Statistical analysis:** Statistical analyses were performed with the software EpiInfo version 6 and SPSS version 17. P values ≤0.05 were considered significant.

**Ethical considerations:** Free and informed consent of participants was obtained. This study received the approval of the institutional review board of the Biomolecular Research Center Pietro Annigoni (CERBA/ LABIOGENE)

## Results

Our study population consisted of 198 blood donors of which 17.7% were women and 82.3% were men. The donors' age ranged between 18 and 56 years with an average of 24.24 ± 6.69 years. More than three quarters (78.3%) of blood donors were from urban areas. The three herpes viruses: EBV, CMV and HHV-6 were diagnosed among blood donors in this study. Of the 198 blood donors tested 18 (9.1%) were positive for at least one of the three viruses. The prevalence of EBV, HHV-6 and CMV was respectively 5.1%; 6.1% and 5.1% (Table 1). No co-infection between EBV / CMV, EBV / CMV and HHV-6 / HHV-6 was observed in this study. While the triple infection EBV / CMV / HHV-6 concerned 3.5% (7/198) of the blood donors. The presence of CMV, EBV and HHV-6 was observed only in blood donors younger than 30 years. A single case of infection with HHV-6 was observed among those aged over 30 years (Table 2). The prevalence of EBV (8, 6% versus 4.3%), CMV (8.6% versus 3.4%) and HHV6 (11.4% versus 4.9%) was higher, respectively in women compared to men, but the observed difference was not statistically significant (Table 2). We did not observe significant differences by comparing the prevalence of human herpes virus between urban and rural areas. However, the prevalence of EBV and CMV was slightly higher in urban than rural areas respectively (5.8% vs 2.3% and 5.2% vs 4.7%); while that of HHV-6 was slightly higher in rural than in urban areas (7.0% vs 5.8%) (Table 2).

**Table 1:** Prevalence of Epstein Barr virus (EBV), cytomégalovirus (CMV) and Herpes virus 6 (HHV-6) in blood donors

	EBV		CMV		HHV-6	
	Number	Percentage (%)	Number	Percentage (%)	Number	Percentage (%)
Negative	188	94.9	188	94.9	186	93.9
Positive	10	5.1	10	5.1	12	6.1
Total	198	100.0	198	100.0	198	100.0

**Table 2:** Infection with cytomégalovirus ( CMV), Epstein Barr virus (EBV) and Herpes virus 6 (HHV-6) as a function of the age, sex and place of blood donation

Characteristics	EBV	CMV	HHV-6	Total
**Age group (years)**				
≤ 30	10 (5.9)	10 (5.9)	11 (6.5)	169
30-40	0 (0.00)	0 (0.00)	1 (4.6)	22
≥ 41	0 (0.00)	0 (0.00)	0 (0.00)	7
**Gender**				
Female	3 (8.6)	3 (8.6)	4 (11.4)	35
Male	7 (4.3)	6 (3.7)	8 (4.9)	163
**Place of blood donation**				
Rural areas	1 (2.3)	2 (4.7)	3 (7.0)	43
Urban areas	9 (5.8)	8 (5.2)	9 (5.8)	155

## Discussion

We show in this study that the overall prevalence of human herpes virus in blood donors was 9.1%. The prevalence of EBV, HHV-6 and CMV was respectively 5.1%; 6.1% and 5.1%. The prevalence of EBV (5.1%) observed in this study among blood donors is similar to the prevalence of 5.4% reported by Tao et al. (2013) [20] on blood donors in the city of Ouagadougou; but is well below the EBV seroprevalence of 20.0% obtained from the study on blood donors from Ghana by Adjei et al. [2] In this study, the prevalence of 5.1% reported in CMV blood donors corroborates the results of 4.3% obtained by Furui et al. in Japan, in 2013 [21]. However, it is lower than the anti-IgG and anti-IgM seroprevalence of 92.2% and 12.2% respectively, observed in Ouagadougou among blood donors [1]. The seroprevalence we obtained from our study is also less than the anti IgG seroprevalence reported by Kothari and al. India in 2002 [22] and by Adjei et al. Ghana in 2006 [23]; who had found a prevalence of 95.0% and 93.3%; respectively. Nevertheless, it remains below the anti-IgM seroprevalence of 0.07% reported by Kumar et al. in 2008, in India. [24] Discrepancies between our results and those of previous studies could be explained by the diagnostic techniques (molecular in our study versus serological in previous studies). Almost all cases of infection with human herpes viruses was observed in blood donors younger than 30 years. Our results are consistent with a previous study which reported that EBV infections, CMV and HHV-6 are more common in young individuals [21, 25, 26]. In our study, we obtained the prevalences of 5.1%; 6.1% and 5.1% respectively for EBV, HHV-6 and CMV, and the EBV infection triple / CMV / HHV-6 concerned 3.5% (7/198) of blood donors. This result is similar to that of Wang et al. in 2010, in China [26] who also reported a rate of 63.6% for EBV / CMV coinfection, with the prevalence of 68.9%, 81.3% for EBV and CMV. Despite the limited sample size, the analysis allows us to report for the first time the prevalence of EBV, CMV and HHV6 in blood donors in Burkina Faso. From the prevalence obtained, we recommend the diagnosis of herpes viruses in all blood donors in order to reduce at best their transmission by blood transfusion.

## Conclusion

This study also reports for the first time, the prevalence of CMV and HHV-6 in Ouagadougou blood donors. A study on a larger sample and including other regions is needed to establish the prevalence of the human herpes viruses in Burkina Faso.

### What is known about this topic

- Immunocompromised persons are more vulnerable to herpes virus infections;
- The herpes virus infections are responsible for cancers;
- The seroprevalence of herpes viruses is very high in Sub-Saharan Africa.

### What this study adds

- This study is among the first conducted in blood donors;
- Herpes Viral infections were higher in women than in men;
- Molecular epidemiology is lower compared to the seroprevalence described in previous studies.
